# Safeguarding pregnant asylum-seekers and refugees during the era of COVID-19

**DOI:** 10.7189/jogh.11.03026

**Published:** 2021-01-30

**Authors:** Tara C Pilato, Faten A Taki, Gunisha Kaur

**Affiliations:** 1 Weill Cornell Medical College, New York, New York, USA; 2 Department of Anesthesia, Weill Cornell Medicine, New York, New York, USA

As it swept across the globe, the novel coronavirus disease 2019 (SARS-CoV-2, or COVID-19) triggered an international public health emergency, preoccupying families, communities, and continents worldwide. Those most affected by this virus are also the most abandoned within their respective host countries: vulnerable populations of migrants, asylum seekers, and refugees. While often scapegoated for introducing new or exotic illnesses, these displaced individuals are likely the first group to be neglected in a national crisis response [1].

COVID-19 has not only magnified health inequities within and across borders, but also further compounded the disparities present in migrant communities, which abide inadequate access to basic health care and sanitary needs in addition to severe overcrowding at baseline. In some countries, public health officials, NGOs, and other aid organizations have been prohibited from entering migrant communities to provide care, out of fear of worsening COVID-19 spread [2]. While the conditions for those living in migrant camps or confined in detention centers are already inhumane, the consequences for pregnant women may be dire.

There are no conclusive studies identifying a clear-cut relationship between pregnancy outcomes and COVID-19 infection. Results of multiple studies remain contradictory. Further, the few studies on pregnant women infected with COVID-19 have multiple limitations [3]. Nonetheless, it is recognized that physiological changes throughout the course of pregnancy alter the coagulation cascade, as well as stress the respiratory and cardiovascular systems; these changes may make pregnant women more susceptible to the deleterious effects of any infection. Most concerningly, however, we know that the provision of antenatal care decreases in public health emergencies. During the 2014 outbreak of Ebola in Liberia, there was a prodigious decline across all touchstones of maternal health. Records of deliveries, stillbirths, neonatal deaths, and maternal mortality declined significantly. Not only was there a steep reduction in health care utilization, as many facilities were closed, but also a serious gap in antenatal care [4].

Pregnant women are an inherently vulnerable group. In numerous countries, reproductive health care is marginalized. The WHO has stated that poorer, disenfranchised women face higher rates of maternal morbidity and mortality, even in countries of high income [5,6]. The American College of Obstetricians and Gynecologists (ACOG) reports that 1 of every 6 battered women first experienced violence while pregnant. If women are enduring ongoing abuse, the severity of intimate partner violence tends to increase during pregnancy [7]. During the ongoing COVID-19 pandemic, it is well-documented that rates of intimate partner violence have surged: for many abused women, nation-wide lockdowns aggravated an already perilous situation [8]. For refugee women, who are known to experience a varied multitude of gender-based violence at baseline, it may be inferred that the rapid spread of COVID-19 worsened similarly cruel circumstances.

**Figure:**
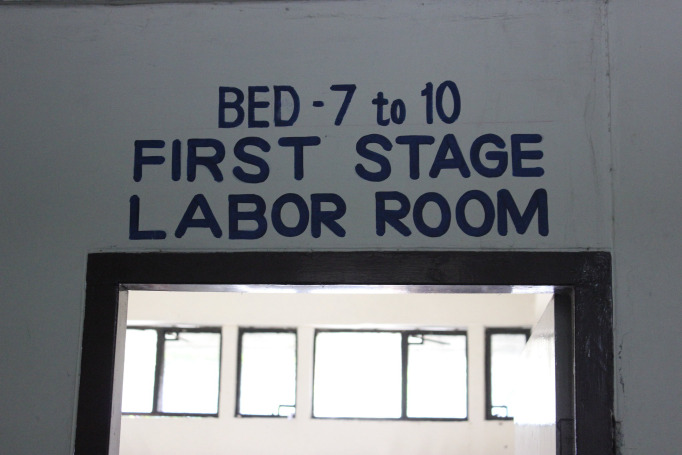
Photo: from Cornell Anesthesia Global Health, used with permission.

Pregnant migrant and refugee women face severe marginalization when seeking antenatal care, a reality only exacerbated by the arrival of the novel coronavirus. In combination with increasing rates of abuse and higher maternal morbidity, these women are living in unhygienic conditions, bereft of basic sanitation or safety. Moreover, as displaced people, they are likely fleeing severe disasters, crises, or persecution – with accompanying trauma from these experiences, or their journey to escape them. Pregnant refugee women may be prohibited by medical costs or be unable to find transportation to clinics; they may have a general mistrust of the health care system, a fear of stigma, or lack fluency in the language of the host country and its clinicians [9].

Highlighting previous insights compiled by Rasmussen et al [3], we propose the following recommendations for pregnant women during the COVID-19 pandemic, tailored for migrant and refugee women [10] (**Table 1**).

**Table 1:** Summary of recommendations

General recommendations for pregnant women with COVID-19 [3]	Migrant and refugee specific considerations [10]
Provide a mask on presentation to the clinic.	Interpreters, whether in-person or virtual, should ALWAYS be available.
Isolate the pregnant patient in a single-occupant room.	Migrant women should be encouraged to discuss their individual circumstances, particulars of their journey to the host country, and cultural practices surrounding their pregnancy. Women should be screened for illnesses endemic to the region and pre-existing conditions that may make them more susceptible to COVID-19 infection.
Consider separating COVID-19 and non-COVID-19 clinical areas, with an assigned medical team for each.	Refugee women may not have had access to resources and information meant to prevent COVID-19 infection. Viral transmission should be explained, and preventative measures (ie, social distancing, covering of the nose and mouth) reviewed in the woman’s native language.
Weigh the benefits of an early epidural with the risks of general anesthesia and intubation, if an urgent Cesarean delivery becomes necessary.	Care should be sensitive, person-centered, and preventative rather than reactionary.
Take into consideration whether to separate a confirmed positive COVID-19 mother from her newborn, to mitigate risk of transmission.	Culturally-sensitive and language-specific information is essential. Training on these topics may be necessary.
-If separated and desirous of breastfeeding, women should practice skin hygiene while expressing breast milk.	Multiple forms of communicating guidelines and recommendations should be used by health care providers, including visual and written aids.
-If not separated, breastfeeding women should wear a face shield or mask.	Migrant and refugee women should always be given sufficient information for their own decision making and empowered to understand their antenatal care.
Newborns should be tested for COVID-19 24 h after birth.	▪

The provision of adequate care to every pregnant woman is of fundamental importance to public health. It is imperative we educate all women to advocate for their maternal needs, as this practice is one of the most effective ways to mitigate maternal morbidity and mortality. In no group is this obligation more urgent than in migrants and refugees, especially during the age of COVID-19. Improving antenatal health care and health outcomes for refugee women will not only prevent pregnancy complications, but will also benefit neonatal health, alleviate overburdened migrant health care systems, and lighten the socioeconomic stressors of mismanaged pregnancy.
